# *In silico* analysis of *GATA4* variants demonstrates main contribution to congenital heart disease

**DOI:** 10.34172/jcvtr.2021.45

**Published:** 2021-11-01

**Authors:** Shiva Abbasi, Neda Mohsen-Pour, Niloofar Naderi, Shahin Rahimi, Majid Maleki, Samira Kalayinia

**Affiliations:** ^1^Cardiogenetic Research Center, Rajaie Cardiovascular Medical and Research Center, Iran University of Medical Sciences, Tehran, Iran; ^2^Zanjan Pharmaceutical Biotechnology Research Center, Zanjan University of Medical Sciences, Zanjan, Iran; ^3^Department of Cardiology, Rajaie Cardiovascular Medical and Research Centre, Iran University of Medical Sciences, Tehran, Iran

**Keywords:** Congenital Heart Disease, GATA4, In Silico Analysis, Transcription Factor

## Abstract

***Introduction:*** Congenital heart disease (CHD) is the most common congenital abnormality and the main cause of infant mortality worldwide. Some of the mutations that occur in the *GATA4* gene region may result in different types of CHD. Here, we report our* in silico* analysis of gene variants to determine the effects of the *GATA4* gene on the development of CHD.

***Methods:*** Online 1000 Genomes Project, ExAC, gnomAD, GO-ESP, TOPMed, Iranome, GME, ClinVar, and HGMD databases were drawn upon to collect information on all the reported *GATA4* variations.The functional importance of the genetic variants was assessed by using SIFT, MutationTaster, CADD,PolyPhen-2, PROVEAN, and GERP prediction tools. Thereafter, network analysis of the GATA4protein via STRING, normal/mutant protein structure prediction via HOPE and I-TASSER, and phylogenetic assessment of the *GATA4* sequence alignment via ClustalW were performed.

***Results:*** The most frequent variant was c.874T>C (45.58%), which was reported in Germany.Ventricular septal defect was the most frequent type of CHD. Out of all the reported variants of *GATA4*,38 variants were pathogenic. A high level of pathogenicity was shown for p.Gly221Arg (CADD score=31), which was further analyzed.

***Conclusion:*** The *GATA4* gene plays a significant role in CHD; we, therefore, suggest that it be accorded priority in CHD genetic screening.

## Introduction

Congenital heart disease (CHD) is the most common congenital malformation and a significant cause of childhood mortality with an estimated prevalence of 1% of infants born each year.^1,2^ Cardiovascular abnormalities are reported in approximately 29% of dead infants. CHD can be caused by variants in different genes whose roles have evolved. The number of genes and variants thereof involved in the CHD pathogenesis has increased, and an accurate determination of the molecular mechanisms of CHD remains particularly challenging due to genetic heterogeneity and incomplete penetrance.^3^ Also extremely complex is the differential diagnosis of CHD in that it is a multifactorial disease encompassing both genetic predisposition and environmental components. ^4^ Thus, it is vitally important to identify disease-causing genetic variants. ^5^ Some CHD-associated genes encode transcription factors such as GATA4, NKX2-5, and TBX5, and a number of gene variants identified in these genes have been associated with cardiac structure and functional impairment. ^1^ GATA-binding factor 4 (GATA4) (OMIM: 600576) is one of the 6-member GATA family of transcription factors: GATA1, GATA2, GATA3, GATA4, GATA5, and GATA6. Amongst GATA-binding proteins, GATA1–3 are expressed in hematopoietic stem cells as significant regulators, whereas GATA4–6 are expressed in different mesoderm- and endoderm-derived tissues such as the heart, the lung, the gonad, the gut, and the liver. ^6^ Variants in the *GATA4*, *GATA5*, and *GATA6* genes have been found in patients with various types of CHD. ^7-9^ GATA proteins comprise 2 conserved zinc finger domains (ZNI and ZNII), which cover various aspects of functions including DNA attachment, GATA4 preservation, and protein-protein and the target DNA sequence interactions. The* GATA4* gene consists of 7 exons located on chromosome 8p23.1-p22. The gene encodes one of the earliest-expressed transcription factors with 442 amino acids and is imperative for normal cardiogenesis. *GATA4*is significantly expressed in embryonic development, with the expression continuing in the adult myocardium. ^10-12^ A rise has been reported in the number of patients with CHD who reach adulthood. ^13^ This transcription factor contains 2 transcriptional activation domains (TAD1 and TAD2); 2 zinc finger domains: 1 at the c-terminal region (CZF) and the other at the n-terminal region (NZF); and 1 nuclear localization signal domain (NLS). ^14^ Variants in the *GATA4* gene are highly associated with different types of CHD, ^15^ including tetralogy of Fallot, ventricular septal defect, atrial septal defect, atrioventricular septal defect, patent ductus arteriosus, dilated cardiomyopathy, and pulmonary valve stenosis. ^14,16-21^

The current literature lacks *in silico* analysis on the variants of the *GATA4* transcription factor and their critical role in the different levels of cardiovascular development. Accordingly, for the first time, we aimed to conduct a comprehensive *in silico* analysis of the effects of *GATA4* alterations associated with CHD.

## Materials and Methods

For the detection of genetic variants in the *GATA4*gene, the following methodology was utilized in the present study:

### Data Collection

The amino acid sequence of the human* GATA4* gene was obtained from the National Center for Biotechnology Information (NCBI; https://www.ncbi.nlm.nih.gov/), based on the human genome assembly GRCh37. Accordingly, the Human Gene Mutation Database (HGMD; http://www.hgmd.cf.ac.uk/ac/index.php), as a strongly reliable database, was employed to identify alterations in the *GATA4*gene. ^22^ Concurrently, all pathogenic/likely pathogenic alterations reported in public access databases were identified. The databases were ClinVar (https://www.ncbi.nlm.nih.gov/clinvar/),^23^ dbSNP (the NCBI database of genetic variation; https://www.ncbi.nlm.nih.gov/snp/), GeneCards (the human gene database; https://www.genecards.org/), ^24^ ExAC (the exome aggregation consortium; http://exac.broadinstitute.org/), ^25^ the 1000 Genomes Project (https://www.internationalgenome.org/), ^26^ gnomAD (the Genome Aggregation Database; http://gnomad.broadinstitute.org/), ^27^ GO-ESP (NHLBI “Grand Opportunity” Exome Sequencing Project; http://evs.gs.washington.edu/EVS/), ^28^ TOPMed (Trans-Omics for Precision Medicine; https://www.nhlbiwgs.org/), ^29^ Iranome (http://www.iranome.ir/), ^30^ and the Greater Middle East (GME) Variome Project (http://igm.ucsd.edu/gme/). ^31^ Moreover, extensive research was carried out through computerized search of PubMed, Scopus, Google Scholar, ScienceDirect, MalaCards (the human disease database), and ResearchGate databases by using the following terms: *GATA4* variants, the clinical importance of the *GATA4* gene, *GATA4*-related disorders, CHD, the pathophysiology of CHD, and the incidence of CHD.

### Frequency

The frequencies of the selected variants were determined using the aforementioned databases. Furthermore, the number of participants and individuals having variations in the studied populations was reported.

### Computational Methods

Given its increasing importance and use to determine the possible effects of genetic variants, computational analysis was employed in the present study. The variants of the *GATA4* gene and their correlations with the molecular pathogenesis of CHD were further explored by predicting the pathogenicity/tolerance of the variants through the following bioinformatics tools: SIFT (Sorting Intolerant from Tolerant; https://sift.bii.a-star.edu.sg/www/SIFT_seq_submit2.html), ^32^ PolyPhen-2 (Polymorphism Phenotyping, version 2; http://genetics.bwh.harvard.edu/pph2/), ^33^ PROVEAN (Protein Variation Effect Analyzer, version 1.1.3; http://provean.jcvi.org/seq_submit.php), ^34^ CADD (Combined Annotation-Dependent Depletion; https://cadd.gs.washington.edu/), ^35^ MutationTaster (http://www.mutationtaster.org/), ^36^ and GERP (Genomic Evolutionary Rate Profiling; http://mendel.stanford.edu/SidowLab/downloads/gerp/). ^37^ All these bioinformatics tools are capable of distinguishing pathogenic from nonpathogenic alterations. Protein sequences in the FASTA format (NM_002052.5), the positions and substitutions of amino acids, and the positions of chromosomes were used as input data. A SIFT score of 0.05 or less is regarded as deleterious, and a SIFT score of greater than 0.05 is considered to signify a tolerated variant. ^32^ PolyPhen-2 results are shown with qualitative levels as benign, possibly damaging, and probably damaging. PolyPhen-2 prediction outputs have a numerical score range of 0 to 1. The cutoff score considered for PolyPhen-2 is 0.5, and variants with scores equal to or greater than 0.5 are predicted to be deleterious. ^33,38^ The cutoff score for PROVEAN is −2.5, and variants equal to or greater than −2.5 are assigned as deleterious. ^34^ Also calculated in the current investigation was the CADD score. All genomic features used to calculate the CADD score via a machine-learning model are summarized into a Phred score with a cutoff point of 20. Disease-causing variants display a high Phred score ( > 20), whereas a low score (<20) signifies less pathogenicity. ^35,39^ MutationTaster, which was applied for all the detected variants in the present study, considers an alteration to be a polymorphism if it is reported as a single-nucleotide polymorphism (SNP) in the HapMap data and the 1000 Genomes Project. Thus, any alteration that could result in premature termination codon and ultimately lead to nonsense-mediated mRNA decay is considered a disease-causing variant. GERP is an evolutionary measurement tool whose results are based on multi-species sequence alignment by comparison with neutral expectation. GERP scores show a reduction in the number of substitutions. Positive scores indicate a substitution deficit, while negative scores show that a site is probably evolving neutrally. ^40^

### GATA4 Network Analysis

The functional association between 2 proteins is the primary purpose of the STRING (Search Tool for the Retrieval of Interacting Genes/Proteins) database. This web-based tool expresses the interaction of proteins in a particular biological function. ^41^ STRING (version 11.0; https://string-db.org/) is used to recognize the known and predicted interactions between the GATA4 protein and other related proteins in a cell. ^42^

### Prediction of Normal and Mutant Protein Structures

Structural and functional differences between wild-type and mutated GATA4 were anticipated by using HOPE (Have [y]Our Protein Explained; https://www3.cmbi.umcn.nl/hope/input/) and ^43^ I-TASSER (Iterative Threading ASSEmbly Refinement; https://zhanglab.ccmb.med.umich.edu/I-TASSER/). ^44-46^ The objective was to analyze a pathogenic variant with a high CADD score. HOPE shows the 3D structural and functional effects of a point mutation in human proteins. The input for this tool is the amino acid sequence of the GATA4 protein and the specific amino acid alteration of the variant. ^43^ The I-TASSER server predicts secondary structures and 3D models through various alignment methods. The accuracy of the formed models is evaluated based on a confidence score (C-score). Predicted models with a C-score of greater than −1.5 are considered to possess a correct topology. I-TASSER predicts the template modeling score (Tm-score) and the root mean square deviation (RMSD). The TM-score ranges between 0 and 1, with higher values specifying better structural models. ^47^

### Phylogenetic Analysis

GATA4 protein sequences from 5 different organisms, namely *Homo sapiens* (humans), *Canis lupus familiaris* (dogs), *Rattus norvegicus* (rats), *Gallus gallus domesticus* (chickens), and *Xenopus laevis* (African clawed frogs), were retrieved from UniProt (the Universal Protein Resource; https://www.uniprot.org/). Afterward, all the GATA4 protein sequences were aligned via the multiple sequence alignment program ClustalW (version 1.83; https://www.genome.jp/tools-bin/clustalw). Thereafter, a phylogenetic tree was built by using ClustalW via the neighbor-joining method. As a result of the multiple sequence alignment, the tree showed scores that represented a sequence distance measure. These values determine the length of the branches, with the length showing the distance between the sequences.

## Results

### Literature Analysis

**Figure 1:**
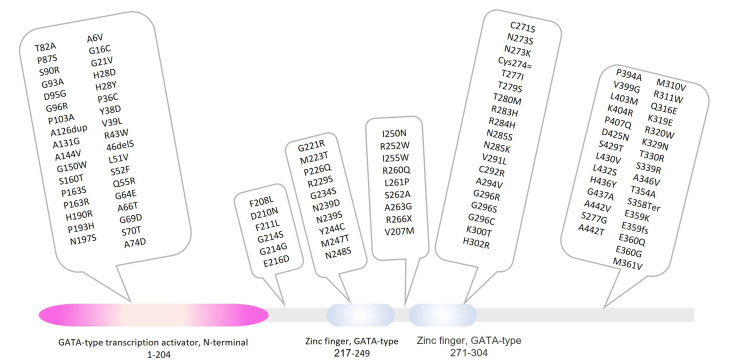
The image shows the distribution of the variations of the *GATA4* gene. The figure demonstrates the 3 main parts of the GATA4 protein, the GATA-type transcription activator, and the zinc finger regions. The numbers beneath the protein structure represent the number of amino acids in the GATA4 amino acid sequence. The reported variations are categorized based on their locations.

Using online databases and publications, we succeeded in finding 110 reported variations in the *GATA4* gene. We also determined the frequency of the gene variants from online resources. The data are depicted in Table 1. The distributions of the reported variants in the different regions of the GATA4 gene are presented in Figure 1.

**Table 1:** Reported frequency of the variants in online databases

**DNA Change**	**Genomic Placement on Chromosome 8**	**1000Genome**	**ExAC**	**GnomAD**	**GO-ESP**	**TOPMED**	**Iranome**	**GME**
c.17C > T	11565838	0.0002	0.00002	0.00003	-	-	-	-
c.46G > T	1156586	0.0002	-	0.00003	-	0.000016	-	-
c.62G > T	11565883	0.0002	0.00004	0.00002588	-	0.000024	-	-
c.82C > G	11565903	-	-	-	-	0.000008	-	-
c.82C > T	11565903	-	-	-	-	0.000008	-	-
c.106C > T	11565927	-	-	-	-	-	-	-
c.112T > G	11565933	-	-	-	-	-	-	-
c.115G > T	11565936	-	0.000007541	0.000007541	-	-	-	-
c.127C > T	11565948	-	0	0.00006	-	0.000056	-	-
c.136-138delTCC	11565957	-	-	-	-	-	-	
c.151C > G	11565972	-	-	-	-	-	-	-
c.155C > T	11565976	-	-	-	-	-	-	-
c.164A > G	11565985	-	-	-	-	-	-	-
c.191G > A	11566012	-	-	-	-	0.000008	-	-
c.196G > A	11566017	-	-	-	-	0.000032	-	-
c.206G > A	11566027	-	-	-	-	-	-	-
c.209G > C	11566030	-	-	-	-	-	-	-
c.221C > A	11566042	-	0.00003625	0.00003625	-	0.000032	-	-
c.244A > G	11566065	-	-	0.00006	-	0.000032	-	-
c.259C > T	11566080	-	-	-	-	-	-	-
c.270C > A	11566091	-	-	-	-	-	-	-
c.278G > C	11566099	-	-	-	-	0.000016	-	-
c.284A > G	11566105	-	-	-	-	-	-	-
c.286G > A	11566107	-	-	-	-	-	-	-
c.307C > G	11566128	-	-	-	-	-	-	-
c.357_359CGC	11566175-11566176	-	-	-	-	-	-	-
c.392C > G	11566213	-	-	0.00003	-	0.000303	-	-
c.431C > T	11566252	-	-	0.0001441	-	0.000016	-	-
c.448G > T	11566269		-	-	-	-	-	-
c.479G > C	11566300	-	-	-	-	-	-	-
c.487C > T	11566308	0.0002	0.0002	0.00003	-	0.000175	-	-
c.488C > G	11566309		0.0002	0.00003	-	0.000008	-	-
c.569A > G	11566390	-	-	-	-	-	-	-
c.578C > A	11566399	-	-	-	-	-	-	
c.590A > G	11566411	-	-	-	-	-	-	-
c.620C > T	11561728	0.2017	-	0.16213	-	0.174583	-	-
c.622T > C	11606433	-	-	-	-	-	-	
c.628G > A	11606439	-	0.00003	-	0.00008	0.000008	-	-
c.631T > C	11606442	-	-	-	-	-	-	
c.640G > A	11606451	-	-	-	-	-	-	-
c.640G > A	11606451	-	-	-	-	-	-	-
c.648C > G	11606459	-	-	-	-	-	-	-
c.661G > A	11606472	-	-	-	-	-	-	-
c.668 T > C	11606479	-	-	-	-	-	-	-
c.677C > A	11606488	-	-	-	-	-	-	-
c.687G > T	11606498	-	-	-	-	-	-	-
c.700G > A	11606511	-	-	-	-	-	-	-
c.715A > G	11606526	-	-	-	-	-	-	-
c.716A > G	11606527	-	-	-	-	-	-	-
c.731A > G	11606542	-	-	-	-	-	-	-
c.740T > C	11606551	-	-	-	-	-	-	-
c.743A > G	11606554	-	0.000016	0.000008	-	-	-	-
c.749T > A	11606560	-	-	-	-	-	-	-
c.754C > T	11606565	-	-	-	-	-	-	-
c.764T > C	11606575	-	-	-	-	-	-	-
c.779G > A	11606590	-	-	0.00003	-	0.000008	-	-
c.782T > C	11606593	-	-	-	-	-	-	
c.783T > G	11606594	-	-	-	-	-	-	-
c.788C > G	11607624	-	-	-	-	-	-	-
c.796C > T	11607632	-	-	-	-	-	-	
c.799G > A	11607635	0.0004	0.000306	0.00029	-	0.000231	-	-
c.812G > C	11607648	-	-	-	-	-	-	-
c.818A > G	11607654	-	-	0.00003	-	-	-	-
c.819C > A	11607655	-	-	-	-	-	-	-
c.822C > T	11607658	0.0018	0.002243	0.00258	0.00308	0.003297	-	-
c.830 C > T	11607666	-	-	0.000004	-	0.000008	-	-
c.835A > T	11607671	-	-	-	-	-	-	-
c.839C > T	11607675	-	-	-	-	-	-	-
c.848G > A	11607684	-	-	-	-	-	-	-
c.851G > A	11607687	-	-	-	-	-	-	-
c.854A > G	11607690	-	-	-	-	-	-	-
c.855T > C	11607691	-	-	-	-	-	-	-
c.871G > C	11607707		-	-	-	-	-	-
c.874T > C	11607710	-	-	-	-	-	-	-
c.881C > T	11607717	-	-	-	-	-	-	-
c.886G > C	11607722	-	-	-	-	-	-	-
c.886G > A	11607722	-	-	-	-	-	-	-
c.886G > T	11607722	-	-	-	-	-	-	-
c.899A > C	11607735	-	-	-	-	-	-	-
c.905A > G	11607741	-	-	-	-	-	-	
c.928A > G	11612573	-	-	-	-	0.000008	-	-
c.931C > T	11612576	-	-	-	-	-	-	-
c.946C > G	11612591	-	0.000008	-	-	-	-	-
c.955A > G	11612600	-	-	-	-	-	-	-
c.958C > T	11612603	-	-	-	-	0.000008	-	-
c.989C > G	11612634	-	-	-	-	-	-	-
c.1017C > A	11614463	-	-	-	-	-	-	-
c.1037C > T	11614483	0.00060	0.00178	0.00124	0.00238	0.00149	-	-
c.1060G > A	11614503	-	-	-	-	-	-	-
c.1074delC	11614520	-	-	-	-	-	-	-
c.1075delG	11614521	-	-	-	-	-	-	-
c.1078G > C	11614524	-	0.000074	-	0.00008	0.000135	-	-
c.1079A > G	11614525	-	-		-	-	-	-
c.1081A > G	11614527	-	-	-	-	-	-	
c.1129A > G	11614575	0.04293	0.09621	0.10632	0.10057	0.08186	-	0.133198
c.1180C > A	11615835	0.0064	0.002522	0.00003	-	0.000080	0.0025	0.003524
c.1196T > G	11615851	-	-	-	-	-	-	-
c.1207C > A	11615862	-	-	-	-	-	-	-
c.1211A > G	11615866	-	-	-	-	-	-	-
c.1220C > A	11615875	0.0012	-	0.00010	0.00113	0.000247	0.000625	0.00503
c.1273G > A	11615928	0.0034	0.002117	0.00003	-	0.000239	0.01188	0.002517
c.1286G > C	11615941	-	-	-	-	-	-	-
c.1288C > G	11615943	-	-	-	-	-	-	-
c.1295T > C	11615950	-	-	-	-	-	-	-
c.1306C > T	11615961	-	-	-	-	-	-	-
c.1310G > C	11615965	-	-	-	-	-	-	-
c.1324G > A	11615979	-	-	-	-	0.000008	-	-
c.1325C > T	11615980	-	-	-	0.00008	0.000104	-	-

### Frequency of the Variants

A wide range of *GATA4* variants has been reported in different countries such as Japan, Australia, the United States, Brazil, Egypt, India, Germany, Lebanon, France, Iran, Italy, and especially China. Precise data on the reported variations and the phenotype condition of the individuals studied in different countries are depicted in Table 2. Genetic alterations in c.1129A > G were reported in 3 countries: China (0.33%), Germany (23%), and Australia (19.04%), with the highest frequency in Germany. Additionally, c.874T > C (45.58%), which was reported in Germany, represented the highest frequency among all the reported variations.

**Table 2:** Frequency of the variants in different populations

**DNA Change**	**Condition** ^1^	**Population (Frequency)**	**CHD Type**	**References**
c.17C > T	-	China (0.2%)	VSD	^48^
c.46G > T	-	China (0.62%)	AF	^49^
c.62G > T	Uncertain significance	China (1%)	ASD	^50,51^
c.82C > G	-	China (0.62%)	AF	^49^
c.82C > T	-	China (2%)	VSD	^52^
c.106C > T	-	China (0.45%)	ASD	^53^
c.112T > G	-	China (0.66%)	AF	^10^
c.115G > T	-	China (0.45%)	DCM	^11^
c.127C > T	Uncertain significance	China (0.62%)	VSD	^54^
c.136-138delTCC	-	China (0.2%)	VSD	^48^
c.151C > G	-	China (1.92%)	TOF	^55^
c.155C > T	Pathogenic	Japan (6.25%)	ASD	^56^
c.164A > G	-	China (0.43%)	VSD	^57^
c.191G > A	-	China (0.38%)	VSD, CTD	^58,59^
c.196G > A	-	China (0.29%)	VSD, PDA, TOF	^59-61^
c.206G > A	-	Australia (0.28%)	VSD	^62^
c.209G > C	-	China (0.76%)	AF	^63^
c.221C > A	-	China (0.26%)	PS	^61^
c.259C > T	-	China (0.55%)	ASD	^50^
c.270C > A	-	China (0.83%)	CHD	^64^
c.278G > C	-	America (0.15%)	ASD	^65^
c.284A > G	-	China (0.83%)	CHD	^64^
c.286G > A	-	China (0.43%)	VSD	^57^
c.307C > G	-	China (0.66%)	AF	^10^
c.357-359CGC	Pathogenic	China (0.2%)	VSD	^48^
c.392C > G	Uncertain significance	Brazil (3.12%)	AVSD	^66^
c.431C > T	-	Japan (0.9%)	PA, ASD	^67^
C.448G > T	-	China (0.26%)	TOF	^61^
c.479G > C	-	China (0.76%)	AF	^63^
c.487C > T	Pathogenic; Uncertain significance	China (0.31%) America (0.93%)	AVSD, VSD, SA+SV, TOF, TGA VSD, PS, TOF, VSD	^48,59,61,68,69^
c.488C > G	Uncertain significance	Australia (0.28%)	VSD	^62^
c.569A > G	-	China (0.45%)	ASD	^53^
c.578C > A	-	Egypt (9.09%)	VSD,VSD, ASD	^70^
c.590A > G	-	China (0.43%)	VSD	^57^
c.620C > T	-	India (3%)	ASD,VSD	^71^
c.622T > C	-	Germany (1.47%)	VSD	^72^
c.628G > A	Uncertain significance	China (0.26%)	AVSD	^61^
c.631T > C	-	Germany (2.9%)	VSD, AVSD	^72^
c.640G > A	-	Germany (1.47%)	VSD	^72^
c.640G > A	-	India (1%)	ASD	^71^
c.648C > G	-	Lebanon (1.66%)	TOF	^73^
c.661G > A	Pathogenic	France (family-based)	CHD	^74^
c.668T > C	-	Germany (1.47%)	VSD	^72^
c.677C > A	-	China (0.45%)	DCM	^11^
c.687G > T	-	Germany (4.41%)	VSD	^72^
c.700G > A	-	Germany (1.47%)	AVSD	^72^
c.715A<G	-	Germany (1.47%)	VSD	^72^
c.716A > G	-	Germany (1.47%)	VSD	^72^
c.731A > G	-	Germany (2.94%)	VSD	^72^
c.740T > C	Uncertain significance	Germany (1.04%)	AF	^75^
c.743A > G	-	Germany (2.94%)	ASD, AVSD	^72^
c.749T > A	-	China (0.26%)	VSD	^61^
c.754C > T	-	Germany (1.47%)	AVSD	^72^
c.764T > C	-	Germany (1.47%)	ASD	^72^
c.779G > A	-	Germany (1.47%)	VSD	^72^
c.782T > C	-	Germany (2.94%)	VSD, ASD	^72^
c.783T > G	-	China (0.45%)	ASD	^53^
c.788C > G	-	China (0.44%)	VSD	^76^
c.796C > T	-	Germany (2.94%)	ASD, AVSD	^72^
c.799G > A	-	China (0.58%)	ASD, CTD	^59,77^
c.812G > C	-	China (0.9%)	DCM	^78^
c.818 A > G	-	Germany (1.47%)	AVSD	^72^
c.819C > A	-	Iran (1)	VSD, ASD	^79^
c.822C > T	Benign; Likely benign; Uncertain significance	Germany (0.97%) Australia (0.28%)	ASD, ASD, DCM, TOF, VSD	^62,80^
c.830C > T	-	Germany (1.47%)	AVSD	^72^
c.835A > T	-	China (0.45%)	DCM	^11^
c.839C > T	Uncertain significance	China (13.33%)	AVSD, ASD	^81^
c.848G > A	-	Germany (1.47%)	AVSD	^72^
c.851G > A	-	France (0.3%)	ASD	^82^
c.854A > G	-	China (1.92%)	TOF	^55^
c.855T > C	-	Germany (1.47%)	AVSD	^72^
c.871G > C	-	China (0.66%)	DCM	^82^
c.874T > C	-	Germany (45.58%)	ASD,VSD, AVSD	^72^
c.881C > T	-	Germany (1.47%) Iran(1)	ASD, CHD	^72,79^
c.886G > C	Pathogenic	China (0.47%)	VSD	^83^
c.886G > A	Pathogenic	America(Family-based) Italy(family-based)	ASD, PVS	^84,85^
c.886G > T	Pathogenic	America (0.93%)	ASD	^68^
c.899A > C	-	China (Family-based)	ASD	^14^
c.905A > G	-	Germany (1.47%)	AVSD	^72^
c.928A > G	Pathogenic	China (Family-based)	ASD	^86^
c.931C > T	-	China(Family-based)	TOF, VSD, ASD, PDA	^87^
c.946C > G	Pathogenic	America (0.31%)	ASD	^65^
c.958C > T	Likely pathogenic; Uncertain significance	Italy (family-based)	ASD	^88^
c.989C > G	Uncertain significant	Japan (0.39%)	PTA,ASD	^89^
c.1017C > A	-	Japan (0.39%)	PA,VSD	^89^
c.1037C > T	-	America (0.93%)	ASD	^68^
c.1060G > A	-	China (1.17%)	ASD	^77^
c.1074delC	-	Japan(family-based)	ASD	^68^
c.1075G > A	Pathogenic	China (0.41%)	VSD	^48^
c.1075delG	Pathogenic	Japan(family-based)	ASD	^56^
c.1079A > G	-	China (0.26%)	VSD	^61^
c.1081A > G	-	Germany (1.47%)	VSD	^90^
c.1129A > G	-	China (0.33%) Germany (23.9%) Australia (19.04%) Iran (45%)	ASD, VSD, AVSD, TOF, PA	^17,59,62,80,91^
c.1180C > A	-	India (1.62%)	AVSD,VSD	^92^
c.1196T > G	-	China (0.45%)	VSD	^53^
c.1207C > A	-	America (0.93%)	ASD	^68^
c.1211A > G	-	China (0.43%)	VSD	^57^
c.1220C > A	Benign; Uncertain significance	China (0.59%) Iran (Family-based)	ASD, AVSD, VSD, TOF, VSD	^17,48,59,69,77,93^
c.1273G > A	Uncertain significance, Pathogenic	America (0.16%)	PA, PS, ASD, TOF, AVSD	^65^
c.1286G > C	-	China (0.2%)	VSD	^48^
c.1288C > G	-	Germany (2.94%)	ASD	^90^
c.1295T > C	-	India (0.32%)	PDA	^92^
c.1306C > T	-	China (8%)	CSDs	^60^
c.1310G > C	-	America (1.28%)	BAV	^94^
c.1324G > A	-	Germany (1.47%)	VSD	^90^
c.1325C > T	Pathogenic	China (0.34%)	VSD	^48^

Abbreviation: AVSD, atrioventricular septal defect; ASD, atrial septal defects; CDH, congenital diaphragmatic hernia; CTD, conotruncal heart defects; CHD,congenital heart disease; CSDS, cardiac septal defects; DCM, dilated cardiomyopathy; DORV, double-outlet right ventricle; DILV, double-inlet left ventricle; LVHT, left ventricular hypertrabeculation; LVNC, left ventricular noncompaction; PA, pulmonary atresia; PA + IVS, pulmonary atresia with interventricular septum; PVS, pulmonary valve stenosis; SA+SV, single atrium with single ventricle; TGA, transposition of the great arteries; TOF, tetralogy of Fallot; TGA, transposition of the great arteries; VSD, ventricular septal defect; PTA, persistent truncus arteriosus; BAV, bicuspid aortic valve; AF, atrial fibrillation; PDA,patent ductus arteriosus; PS, pulmonary stenosis ^1^According to ClinVar

### Bioinformatics

The results of the identification and analysis of the variations via online prediction tools are shown in Table 3. Out of the 110 substitutions identified, PROVEAN predicted 55 variations to be deleterious and 50variations to beneutral. (Five variations were not available.) SIFT predicted 62 alterations to be damaging and 33 variations to be tolerated. (Fourteen variations were not available.) PolyPhen-2 defined 25 variations as benign, 18 as possibly damaging, and 59 as probably damaging. (Eight variations were not available.) MutationTaster predicted 82 disease-causing variations and 11 polymorphisms. (Seventeen variations were not available.) The maximum CADD score (Phred score = 53) was shown by c.796C > T R266X, indicating high pathogenicity, while c.196G > A, A66T showed the lowest CADD score (Phred score = 0.009). As a result, among the 110 substitutions, 38 were predicted to be deleterious by PROVEAN, SIFT, PolyPhen-2, and MutationTaster.

In this study, c.1075G > A indicated the highest GERP score (5.83), which represents 4.83 fewer substitutions than was expected. No negative GERP scores were reported for these variations.

**Table 3:** In silico analysis of GATA4 variations

**DNA Change**	**Protein Change**	**Variant Type**	**dbSNP**	**HGMD**	**CADD** ^1^	**MutationTaster**	**PolyPhen** ^2^ ** (Score)**	**PROVEAN** ^3^	**SIFT** ^4^ ** (Score)**	**GERP**
c.17C > T	A6V	Missense	rs199922907	CM086821	24.48	DC	PRD (0.986)	NE	DE (0.01)	NA
c.46G > T	G16C	Missense	rs533331682	CM117802	23	POLYMORPHISM	PRD (1.000)	NE	TO (0.1)	NA
c.62G > T	G21V	Missense	rs202213149	CM107596	24.4	DC	PRD (0.972)	NE	DE (0.02)	NA
c.82C > G	H28D	Missense	rs1406275331	CM117803	25	DC	PRD (0.993)	DE	DE (0)	NA
c.82C > T	H28Y	Missense	rs1406275331	CM0910178	24	DC	PRD (0.993)	DE	DE (0)	NA
c.106C > T	P36C	Missense	-	CM1313746	25.2	DC	PRD (1)	DE	DE (0)	NA
c.112T > G	Y38D	Missense	-	CM123513	26	DC	PRD (0.998)	DE	DE(0)	NA
c.115G > T	V39L	Missense	rs1139241	CM147377	24	DC	PRD (0.958)	NE	DE (0)	NA
c.127C > T	R43W	Missense	rs387906770	CM119519	25	DC	PRD (1)	DE	DE (0)	NA
c.136 e138delTCC	46delS	deletion	-	-	-	-	-	NE	-	NA
c.151C > G	L51V	Missense	-	CM1312064	23.6	DC	PRD (0.977)	NE	DE (0.01)	NA
c.155C > T	S52F	Missense	rs104894074	CM1312064	25.6	DC	PRD (0.975)	DE	DE (0)	NA
c.164A > G	Q55R	Missense		CM125062	22.9	DC	POD (0.586)	NE	DE (0.01)	NA
c.191G > A	G64E	Missense	rs1249347695	CM107237	11.21	POLYMORPHISM	BENIGN (0.392)	NE	TO (0.99)	NA
c.196G > A	A66T	Missense	rs1139244	CM1010269	0.009	DC	BENIGN (0)	NE	TO (0.58)	NA
c.206G > A	G69D	Missense		CM109056	-	POLYMORPHISM	BENIGN (0.157)	NE	TO (0.46)	NA
c.209G > C	S70T	Missense	-	CM115165	10.71	DC	BENIGN (0.001)	NE	TO (0.46)	NA
c.221C > A	A74D	Missense	rs1258064099	CM1010265	19.96	POLYMORPHISM	PRD (0.997)	NE	TO (0.14)	NA
c.244A > G	T82A	Missense	rs961114777	-	12.10	POLYMORPHISM	BENIGN (0)	-	TO (0.38)	NA
c.259C > T	P87S	Missense	-	CM107597	-	-	PRD (0.977)	NE	TO (0.14)	NA
c.270C > A	S90R	Missense	-	CM104917	-	-	BENIGN (0.440)	NE	DE (0.04)	NA
c.278G > C	G93A	Missense	rs56191129	CM076206	19.85	DC	POD (0.943)	NE	TO(0.09)	NA
c.284A > G	D95G	Missense	-	CM104918	-	-	BENIGN (0)	NE	TO (0.06)	NA
c.286G > A	G96R	Missense	-	CM1213107	-	POLYMORPHISM	BENIGN (0.012)	NE	TO (0.06)	NA
c.307C > G	P103A	Missense	-	CM123514	19.50	DC	BENIGN (0.001)	NE	TO (0.7)	NA
c.357_359CGC	A126dup	Duplication	rs1182566703	-	-	-	-	NE	-	NA
c.392C > G	A131G	Missense	rs1013984246	-	18.4	POLYMORPHISM	BENIGN (0.002)	NE	TO (0.66)	NA
c.431C > T	A144V	Missense	rs1308945507	CM161974	14.22	POLYMORPHISM	POD (0.727)	NE	TO (0.16)	NA
c.448G > T	G150W	Missense	rs1024075653	CM1010266	26.0	DC	PRD (0.997)	DE	DE (0)	NA
c.479G > C	S160T	Missense	rs1358565879	CM115166	23.5	DC	POD (0.891)	NE	TO (0.35)	NA
c.487C > T	P163S	Missense	rs387906769	CM076201	22.1	DC	POD (0.669)	NE	NA	NA
c.488C > G	P163R	Missense	rs540578824	CM109057	25.4	DC	PRD (0.973)	DE	TO (0.42)	NA
c.569A > G	H190R	Missense	-	CM1313747	24	DC	PRD (0.988)	DE	DE (0)	NA
c.578C > A	P193H	Missense	-	-	24.2	DC	POD (0.921)	NE	DE (0.05)	NA
c.590A > G	N197S	Missense	-	CM125063	15.18	POLYMORPHISM	BENIGN (0.009)	NE	TO (0.65)	NA
c.620C > T	-	5′ UTR	rs61277615	-	8.145	-	-	-	-	NA
c.622T > C	F208L	Missense	--	-	20.6	DC	BENIGN (0.071)	NE	TO (1)	NA
c.628G > A	D210N	Missense	rs377673676	CM1010267	32	DC	PRD (0.996)	DE	NA	5.08
c.631T > C	F211L	Missense	-		22.4	DC	BENIGN (0.005)	NE	TO (0.47)	NA
c.640G > A	G214G	Synonymous	-	CM051488	24.3	-	-	NE	TO (1)	NA
c.640G > A	G214S	Missense	-		24.3	DC	POD (0.921)	DE	TO (0.442)	NA
c.648C > G	E216D	Missense	-	CM061008	-	-	PRD (0.999)	DE	DE (0)	NA
c.661G > A	G221R	Missense	rs398122402	CM110562	32	DC	PRD (0.999)	DE	DE (0)	NA
c.668T > C	M223T	Missense	-	-	23.6	DC	BENIGN (0.126)	DE	TO (0.32)	NA
c.677C > A	P226Q	Missense	-	CM147378	25.3	DC	PRD (1)	DE	DE (0)	NA
c.687G > T	R229S	Missense	-	-	24.2	DC	PRD (0.998)	DE	DE (0)	3.83
c.700G > A	G234S	Missense	-	-	28.1	DC	PRD (1)	DE	DE (0)	5.61
c.715A > G	N239D	Missense	-	-	27.3	DC	PRD (0.995)	DE	NA	NA
c.716A > G	N239S	Missense	-	-	25.8	DC	PRD (1)	DE	DE (0)	NA
c.731A > G	Y244C	Missense	-	-	28.9	DC	PRD (1)	DE	DE (0)	NA
c.740T > C	M247T	Missense	rs1131691325	CM104219	25.5	DC	POD (0.890)	DE	DE (0)	NA
c.743A > G	N248S	Missense	rs749360828	-	25.9	DC	PRD (0.994)	DE	DE (0)	NA
c.749T > A	I250N	Missense	-	CM1010268	27.8	DC	PRD (0.994)	DE	DE (0.01)	NA
c.754C > T	R252W	Missense	-	CM131318	31	DC	PRD (1)	DE	DE (0)	NA
c.764T > C	I255T	Missense	-	-	25	DC	POD (0.748)	DE	DE (0)	NA
c.779G > A	R260Q	Missense	rs1245034279	-	27.9	DC	PRD (0.979)	DE	DE (0.01)	NA
c.782T > C	L261P	Missense	-	-	26.2	DC	POD (0.653)	DE	DE (0)	NA
c.783T > G	S262A	Missense	-	CM1313748	-	-	BENIGN (0.255)	NE	TO (0.2)	NA
c.788C > G	A263G	Missense	-	CM128406	24.5	DC	BENIGN (0.449)	NE	DE (0.04)	NA
c.796C > T	R266X	Nonsense	-	--	53	DC	-	NA	NA	NA
c.799G > A	V267M	Missense	rs116781972	CM068343	24.9	DC	BENIGN (0.401)	NE	TO (0.09)	NA
c.812G > C	C271S	Missense	-	-	27.7	-	PRD (1)	DE	DE (0)	NA
c.818A > G	N273S	Missense	rs1340083717	-	25.9	DC	PRD (1)	DE	DE (0)	NA
c.819C > A	N273K	Missense	-	-	25.3	-	PRD (1)	DE	DE (0)	NA
c.822C > T	Cys274 =	synonymous	rs55980825	-	11.33	DC	-	NE	-	4.51
c.830C > T	T277I	Missense	rs1236909953	-	27	DC	POD (0.770)	DE	DE (0)	NA
c.835A > T	T279S	Missense	-	-	26.3	-	PRD (0.999)	DE	DE (0)	NA
c.839C > T	T280M	Missense	rs387906771	CM104913.	29	DC	PRD (1)	DE	DE (0)	NA
c.848G > A	R283H	Missense	rs180765750	-	31	DC	PRD (1)	DE	DE (0)	NA
c.851G > A	R284H	Missense	-	CM160385	31	DC	PRD (1)	DE	DE (0)	NA
c.854A > G	N285S	Missense	-	CM1312065	24.4	DC	POD (0.858)	DE	DE (0.02)	NA
c.855T > C	N285K	Missense	-	CM051504	0.667	DC	PRD (0.999)	DE	DE (0)	NA
c.871G > C	V291L	Missense	-	CM141469	25.4	DC	PRD (0.997)	DE	DE (0)	NA
c.874T > C	C292R	Missense	-	CM051505	27	DC	PRD (1)	DE	DE (0)	NA
c.881C > T	A294V	Missense	-	CM051506	28.2	DC	PRD (1)	DE	DE (0)	NA
c.886G > C	G296R	Missense	rs104894073	CM114666	27.9	DC	PRD (1)	DE	NA	NA
c.G886A	G296S	Missense	rs104894073	CM031685	27.8	DC	PRD (1)	DE	NA	NA
c.886G > T	G296C	Missense	rs104894073	CM076203	29.2	DC	PRD (1)	DE	DE (0)	NA
c.899A > C	K300T	Missense	-	CM160006	24.6	DC	PRD (1)	DE	DE (0)	NA
c.905A > G	H302R	Missense	-	CM051507	24.6	DC	PRD (0.962)	DE	DE (0)	NA
c.928A > G	M310V	Missense	rs387906772	CM102095	26.4	DC	POD (0.934)	DE	DE (0)	NA
c.931C > T	R311W	Missense	-	-	-	DC	PRD (0.999)	DE	DE(0)	NA
c.946C > G	Q316E	Missense	rs56298569	CM076200	26.6	DC	PRD (0.996)	DE	DE (0)	NA
c.955A > G	K319E	Missense	-	CM140184	29.2	DC	PRD (0.991)	DE	DE (0.01)	NA
c.958C > T	R319W	Missense	rs1282433424	CM106844	33	DC	PRD (1)	DE	DE (0)	NA
c.989C > G	T330R	Missense	-	CM123286	23.5	DC	BENIGN (0.048)	DE	TO (0.14)	NA
c.1017C > A	S339R	Missense	rs1042942931	-	20.9	DC	BENIGN (0.93)	NE	DE (0.03)	NA
c.1037C > T	A346V	Missense	rs115372595	CM076205	14.12	POLYMORPHISM	BENIGN (0.112)	NE	TO (0.28)	3.93
c.1060G > A	T354A	Missense	-	CM107551	-	-	BENIGN (0)	NE	TO (0.21)	NA
c.1074delC	S358X	Nonsense	-	-	-	-	-	-	-	NA
c.1075G > A	E359K	Missense	rs368489876	CM086820	25.2	DC	PRD (1)	NE	NA	5.83
c.1075delG	E359fs	Deletion	rs1585703301	-	-	-	-	-	-	NA
c.1078G > C	E360Q	Missense	rs141808522	-	24.6	DC	PRD (0.985)	NE	NA	5.83
c.1079A > G	E360G	Missense	-	CM1010264	25.9	DC	PRD (0.985)	NE	TO (0.37)	NA
c.1081A > G	M361V	Missense	-	-	17.29	DC	POD (0.664)	NE	TO (0.49)	NA
c.1129A > G	S377G	Missense	rs3729856	CM164458		-	BENIGN (0)	NE	TO (0.56)	0.906
c.1180C > A	P394A	Missense	rs200319078	CM119355	17.05	POLYMORPHISM	BENIGN (0)	NE	TO (0.39)	2.670
c.1196T > G	V399G	Missense	-	CM1313749	20.7	DC	BENIGN (0.131)	NE	TO (0.53)	NA
c.1207C > A	L403M	Missense	-	CM076202	25	DC	PRD (0.997)	NE	TO (0.06)	NA
c.1211A > G	K404R	Missense	-	CM125064	26.4	DC	PRD (0.996)	NE	DE (0)	NA
c.1220C > A	P407Q	Missense	rs115099192	CM086819	25.8	DC	POD (0.675)	DE	DE (0.04)	4.780
c.1273G > A	D425N	Missense	rs56208331	CM076207	29	DC	PRD (0.970)	DE	DE (0.01)	5.180
c.1286G > C	S429T	Missense	-	CM086818	23.18	DC	POD (0.646)	NE	DE (0.04)	NA
c.1288C > G	L430V	Missense	-	-	24.8	DC	PRD (0.990)	NE	DE (0)	NA
c.1295T > C	L432S	Missense	-	CM119354	27.98	DC	PRD (0.998)	DE	DE (0)	NA
c.1306C > T	H436Y	Missense	-	CM095707	26.08	DC	POD (0.851)	NE	DE (0)	NA
c.1310G > C	G437A	Missense	-	CM149081	23.5	DC	POD (0.787)	NE	DE (0)	NA
c.1324G > A	A442T	Missense	rs1270266865	-	26.8	DC	PRD (0.996)	NE	DE (0)	NA
c.1325C > T	A442V	Missense	rs146017816	-	27.1	DC	PRD (0.999)	NE	DE (0)	5.18

All GATA4 variants are reported based on the NCBI nucleotide (NM_002052.5) and protein (NP_002043.2) sequences (NG_008177.2). ^1^ CADD, Phred ≤ 20: Neutral; Phred > 20: Damaging; ^2^ PolyPhen-2, score = 0-0.15: Benign; score = 0.15-0.85: Possibly damaging; score = 0.85-1: Probably damaging; ^3^ PROVEAN, score ≤ -2.5: Deleterious; score > -2.5: Neutral; ^4^ SIFT, score ≤ 0.05: Deleterious; score > 0.05: Tolerable; TO: Tolerable; DE: Deleterious; NE: natural, DC: Disease-causing; NA: Not available. PRD: Probably damaging; POD: Possibly damaging

### Protein-Protein Interaction Network Analysis

**Figure 2:**
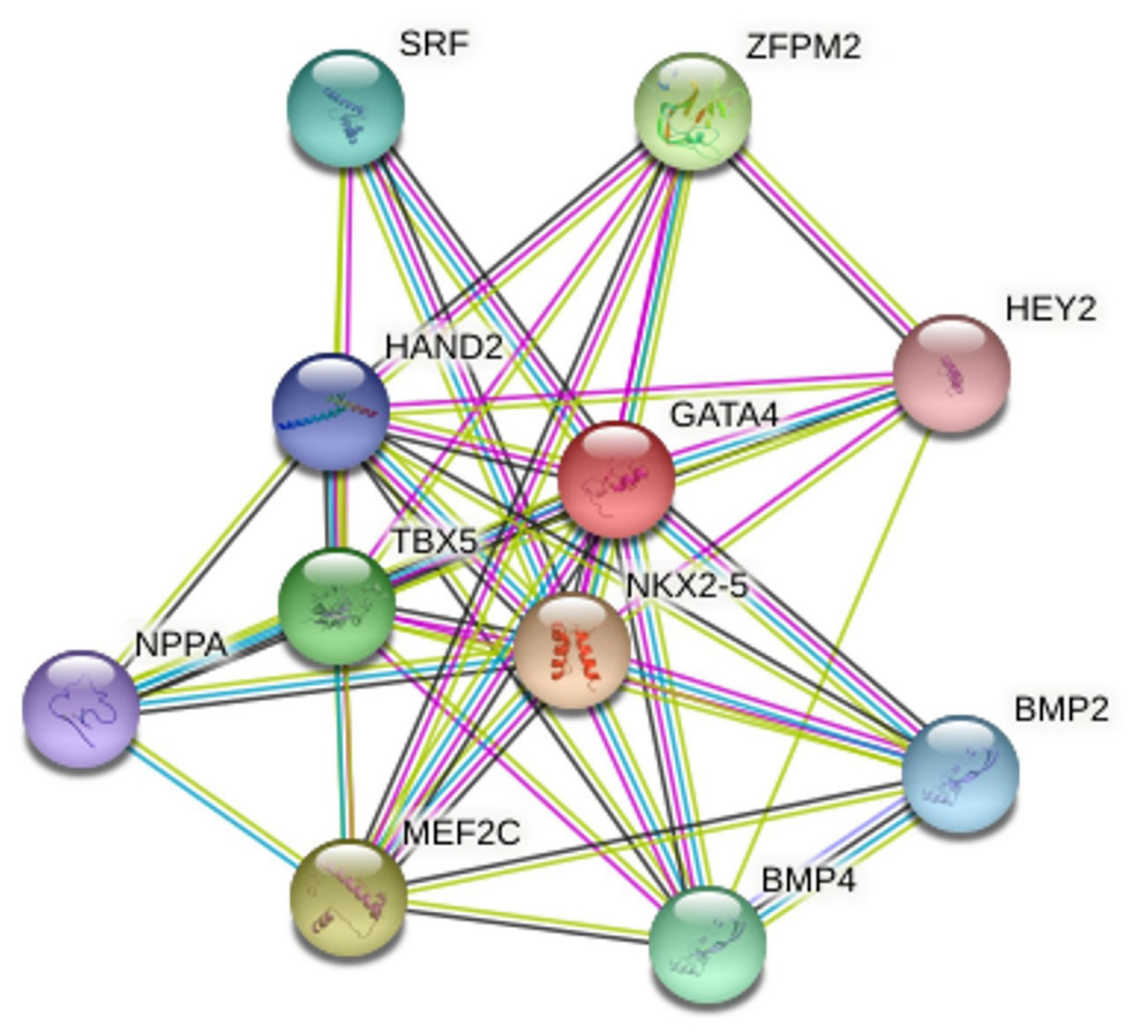
The image presents the STRING protein-protein interaction analysis. The network contains 11 nudes and 41 edges. The edges are represented with various colors, with each color indicating protein-protein associations. The GATA4 protein and its functional interactions with 11 other proteins display possible effects on each other.

As is illustrated in Figure 2, STRING, version 11.0, demonstrated that 11 proteins (GATA4, NKX2-5, MEF2C, ZFPM2, TBX5, BMP4, SRF, BMP2, HAND2, NPPA, and HEY2) and 41 edges (protein-protein associations) grouped to create a protein network.

*NKX2-5*: Homeobox protein NKX2-5, *MEF2C*: Myocyte-specific enhancer factor 2C, *ZFPM2*: Zinc finger protein *ZFPM2*, *TBX5*: T-box transcription factor,* BMP4*: Bone morphogenetic protein 4, SRF: Serum response factor, BMP2: Bone morphogenetic protein 2, HAND2: Heart- and neural crest derivatives-expressed protein 2, NPPA: Natriuretic peptides A, HEY2: Hairy/enhancer-of-split related with YRPW motif protein 2.

### Differences Between the Wild-Type GATA4 Protein and the Mutant Model

**Figure 3:**
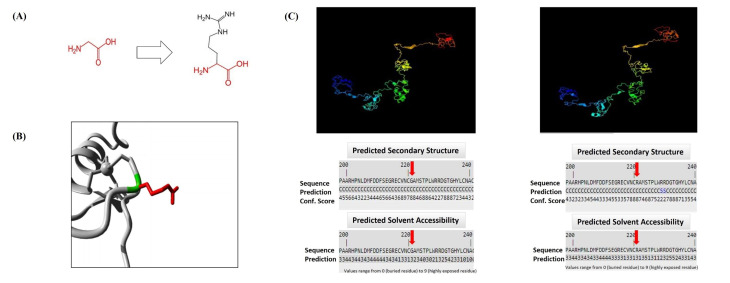
**A)** The image presents the schematic structure of a normal amino acid on the left (glycine) and a mutant one on the right (arginine) at position 221 of the GATA4 protein. The red parts show the similar parts of the amino acids (the backbone), and the black part shows the unique part of the amino acids (the side chain). This picture illustrates the structural differences between the 2 amino acids. The G221R alteration is shown by HOPE. **B)** A photograph generated by HOPE shows that the G221R variation affects the structure of the GATA4 protein. The green color shows the wild-type residue (glycine), and the red color represents the mutant residue (arginine).** C)** I-TASSER shows the secondary and 3D structure, as well as the predicted solvent accessibility, of the normal (left) and G221R mutant (right) of the GATA4 protein.

In this study, the effects of the predicted disease-causing p.Gly221Arg variant in *GATA4* with the CADD Phred score of 31 were further analyzed. The variant, p.Gly221Arg, with a high level of pathogenicity is a heterozygous missense variant in the conserved N-terminal zinc finger of *GATA4*. ^74^ HOPE results showed the alteration of glycine to arginine at position 221 (G221R, CADD Phred = 31). The size, charge, and hydrophobicity value of the 2 residues, as well as the differences between them, are presented in Figure 3A. The mutant residue showed a larger size, with a positive charge, while the wild-type protein charge was neutral. Furthermore, arginine was more hydrophobic than was glycine. These differences in amino acid features could affect the zinc finger site of the protein and its function. Accordingly, this change in the *GATA4* sequence might result in the conformation of the protein and exert negative influences on the structure of the protein in this specific residue (Figure 3B).I-TASSER produced 3D structures of *GATA4* in 5 models with different C-scores. A model with a C-score of −0.5, an estimated TM-score of 0.65, and an estimated RMSD of 8.2 Å was selected. Hence, the findings proved that the solubility of the mutant protein was similar to that of the wild-type one, with a score of 3 (Figure 3C).

### GATA4 Protein Sequence Alignment and the Phylogenetic Tree

**Figure 4:**
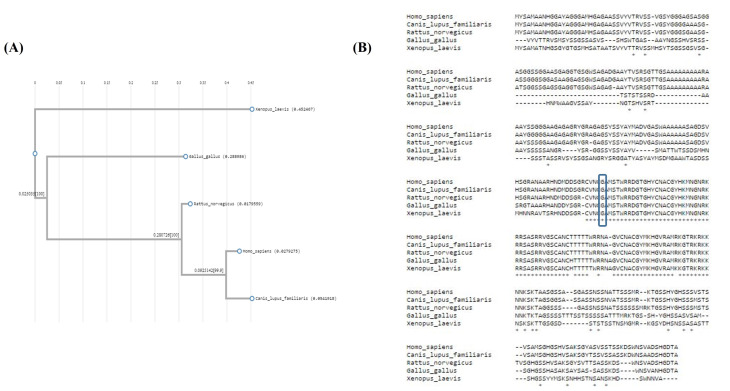
**A)** The phylogenetic tree is constructed by CLUSTALW implementing the neighbor-joining method for GATA4 in *Homo sapiens* (humans), *Rattus norvegicus* (rats), *Canis lupus familiaris* (Dogs), *Xenopus laevis* (African clawed frogs), and *Gallus gallusdomesticus* (chickens). The length of the horizontal lines shows the evolutionary distance between each organism based on the GATA4 protein sequence. (**B)** The results of the CLUSTALW multiple-sequence-alignment program show the conservation of the GATA4 G221 position among the different organisms. The identical (*), conserved (:), and semiconserved (.) residues are specified. This position is highly conserved among the different species.

According to the phylogenetic tree generated by ClustalW, the human GATA4 protein had the closest homology with that of *Canis lupus familiaris* (dogs). Further, the most distant orthologue was *Xenopus laevis* (African clawed frogs) (Figure 4A). The results of the multiple-alignment sequencing of the species are illustrated in Figure 4B.

## Discussion

CHD is the most frequent congenital abnormality and the major cause of infant mortality the world over. GATA4, a transcription factor with 2 zinc finger domains, has been reported to play an essential role in embryogenesis and cardiac development. ^90^ The *GATA4* gene is reported to modulate heart hypertrophy in adults. ^95^ The number of studies seeking to explicate the correlation between *GATA4* variants and CHD occurrence is on the rise. Indeed, recent studies have identified several novel variants in the *GATA4* gene with potential roles in CHD development. ^17^

CHD is very heterogeneous, and the etiology of the majority of cases remains greatly unknown. Both genetic and environmental factors contribute to CHD. ^96^ Therefore, the elucidation of the pathogenesis and differential diagnosis of the disease requires the identification of not only the disease-causing or susceptibility genes but also new genetic variants associated with the different types of CHD. Research has linked several genes to CHD, with *NKX2-5*, *TBX5*, and *GATA4* comprising the most studied transcription factor genes. ^15^ These genes interact during embryonic development, and they are involved in the regulation of cardiogenesis and embryonic heart development. ^97^ Protein-protein interactions between transcription factors play a vital role in biological systems. The results concerning GATA4 protein interactions, generated by STRING, showed that 11 proteins (GATA4, NKX2-5, MEF2C, ZFPM2, TBX5, BMP4, SRF, BMP2, HAND2, NPPA, and HEY2) grouped in a network. GATA4 and NKX2-5 transcription factors are critical to cardiomyocyte hypertrophy; thus, single-point variants could create an imbalance in the interaction between these proteins. ^12^ *GATA4* has been shown to interact with *HAND2* to modulate the transcription of the downstream gene by binding to the conserved GATA-binding sites of the *HAND2* promoter. ^98^ NKX2-5, as a central regulator of many aspects of heart development, interacts with SRF and GATA4 to promote the expression of the cardiac sarcomeric protein gene. ^99^ Mutations in the *ZFPM2* gene, which encodes the FOG2 protein (a transcription regulator of the GATA family members), disrupt the interaction with GATA4 or the nucleosome remodeling and deacetylation (NuRD) complex and, thus, lead to CHD. ^100-103^ Loss-of-function mutation in the *MEF2C* gene, which encodes a transcription factor required for normal cardiovascular development, is associated with increased vulnerability to CHD in humans. ^104^ MEF2C, TBX5, and GATA4 can induce cardiomyocyte differentiation and directly reprogram endogenous cardiac fibroblasts into functional cardiomyocytes. ^105^ Remarkably, BMP2 and BMP4 are vital for cardiogenesis in that they induce the expression of NKX2-5 and GATA4 transcription factors. These 2 genes play a significant role during the initial induction of cardiogenesis. Nevertheless, no association between BMP2 and BMP4 genetic variations (rs1049007, rs235768, and rs17563) and the risk of CHD was reported by Li FF et al. ^106^ Variations in the *NPPA* gene, which encodes the ANP precursor, are correlated with hypertension, stroke, coronary artery disease, and heart failure. ^107^ The HEY2 transcription factor plays an important function in mammalian heart development. Three non-synonymous variations, namely c.286A > G (p.Thr96Ala), c.293A > C (p.Asp98Ala), and c.299T > C (p.Leu100Ser), were reported to affect the second helix of HEY2 in the diseased cardiac tissues of 2 cases with atrioventricular septal defect, suggesting its possible function in the regulation of ventricular septation in humans. ^108^ Somatic mutations were identified in *NKX2-5* and its molecular partners, TBX5 and GATA4, as well as the transcription factor HEY2, in formalin-fixed tissues taken from a collection of hearts with atrial septal defect, ^109^ ventricular septal defect, and atrioventricular canal defect. ^90,108,110-112^

The *GATA4* missense variation (p.G221R), on which we focused in the present study, was identified in three 46, XY DSD patients from a family of French origin. The in vitro assays in that investigation demonstrated the failure of the p.G221R mutant protein to bind to FOG2, which is required for gonad formation. Furthermore, the mutant protein failed to transactivate the anti-Müllerian hormone promoter. ^74^

Some variants of *GATA4* investigated in the present study have been previously analyzed for genotype-phenotype correlations. These investigations evaluated families manifesting those variations associated with different CHD types.

Lourenço D et al ^74^ reported the G221R variant in 5 members of a family with cardiac anomalies including atrial septal defect, tetralogy of Fallot, and congenital cyanotic heart disease.

In a study conducted by Garg V et al, ^84^ the c.886G > A (G296S) variation of *GATA4* was stated in 13 affected members with atrial septal defect in a family with 5 generations. The authors also reported the E359del variation of *GATA4* in 5 members of another family with the autosomal dominant transmission of atrial septal defect in 4 generations, indicating *GATA4* as a genetic cause of atrial septal defect.

Sarkozy et al ^85^ detected the G296S variation of *GATA4* in 2 members of 1 family and 3 members of another family diagnosed with atrial septal defect.

Chen J et al ^14^ recognized the *GATA4* c.899A > C (K300T) substitution in 10 members of a family: 8 affected members with severe symptoms (7 patients with atrial septal defect and 1 patient with ventricular septal defect) and 2 unaffected members. The K300T substitution lessens the transcription of the *GATA4* target gene by harming the DNA-binding activity of GATA4.

Yu Chen et al ^86^ identified the c.928A > G (M310V) variant located in the NLS region of *GATA4* in all patients of a 3-generation family with atrial septal defect. The variant reduces the transcriptional activity of the GATA4 protein and may disturb the interaction between GATA4 and TBX or NKX2-5.

A genetic investigation conducted by E. D’Amato et al ^88^ reported the R319W variation in 3 members of a family: the proband and the proband’s sister, both diagnosed with atrial septal defect, and the proband’s father, who was considered not affected.

Rajagopal et al ^68^ studied 107 probands with cardiac abnormalities and identified the c.886G > T (G296C) variant in a proband with atrial septal defect and pulmonary stenosis. They also reported the substitution in the proband’s father with persistent left superior vena cava to the coronary sinus. The G296S variation resulted in a reduction in GATA4 DNA-binding activity and disrupted binding to the transcription factor TBX5. Also in their study, the c.1207C > A (L403M) variant was identified in a proband with a hypoplastic right ventricle and sinus venosus atrial septal defect. Their results also demonstrated the c.487C > T (P163S) and c.1037C > T (A346V) variants in probands with endocardial cushion defect. Additionally, a missense variation, c.931C > T (R311W), in *GATA4* was identified in a pedigree spanning 3 generations with 7 members diagnosed with CHD. All the affected members presented different cardiac phenotypes, including tetralogy of Fallot, ventricular septal defect, atrial septal defect, and patent ductus arteriosus, indicating that the same genetic alteration could lead to different subtypes of CHD. ^87^

In the present study, we filtered the literature and online databases for the pathogenic variants of the *GATA4* gene. Our search yielded 210 variants; nonetheless, we excluded 100 of these variants due to a dearth of information and continued the study with 110 variations. After analyzing the frequency distributions of all the variants, we employed computational tools with different algorithms to predict the pathogenicity of the variants. As is shown in Table 3, our *in silico* analysis using MutationTaster, PolyPhen, PROVEAN, and SIFT revealed 38 pathogenic genetic variations. Our findings may broaden the spectrum of the known *GATA4* genetic variations associated with different types of CHD.

## Conclusions

Several gene deficiencies could contribute to the pathogenesis of CHD. In this study, we drew upon different *in silico* predictive tools for the analysis of the variants of the *GATA4* gene. The most frequent variant was c.874T > C (45.58%), and the most frequent type of CHD was ventricular septal defect. Out of all the reported variants of *GATA4*, 38 variants were pathogenic. The p.Gly221Arg variant (CADD score = 31) showed a high level of pathogenicity. All the identified pathogenic variations in *GATA4* could assist in the rapid identification and better understanding of the mechanisms underlying CHD.

## Acknowledgments

The authors wish to thank the Rajaie Cardiovascular Medical and Research Center, Tehran, Iran, and Zanjan University of Medical Sciences, Zanjan, Iran.

## Competing Interests

None declared.

## Ethical Approval

Not applicable.

## Funding

This research was funded by Rajaie Cardiovascular Medical and Research Center, Tehran, Iran, and Zanjan University of Medical Sciences, Zanjan, Iran.

## References

[1] Williams K, Carson J, Lo C (2019). Genetics of congenital heart disease. Biomolecules.

[2] Abdul Samad F, Suliman BA, Basha SH, Manivasagam T, Essa MM (2016). A comprehensive in silico analysis on the structural and functional impact of SNPs in the congenital heart defects associated with NKX2-5 gene-a molecular dynamic simulation approach. PLoS One.

[3] Edwards JJ, Gelb BD (2016). Genetics of congenital heart disease. Curr Opin Cardiol.

[4] Ferencz C, Loffredo CA, Rubin JD, Magee CA. Perspectives in Pediatric Cardiology. Armonk, New York: Futura Publishing Company; 1997.

[5] Shabana NA, Shahid SU, Irfan U (2020). Genetic contribution to congenital heart disease (CHD). Pediatr Cardiol.

[6] Martinez de LaPiscina I, de Mingo C, Riedl S, Rodriguez A, Pandey AV, Fernández-Cancio M (2018). GATA4 variants in individuals with a 46,XY disorder of sex development (DSD) may or may not be associated with cardiac defects depending on second hits in other DSD genes. Front Endocrinol (Lausanne).

[7] Kodo K, Nishizawa T, Furutani M, Arai S, Yamamura E, Joo K (2009). GATA6 mutations cause human cardiac outflow tract defects by disrupting semaphorin-plexin signaling. Proc Natl Acad Sci U S A.

[8] Jiang JQ, Li RG, Wang J, Liu XY, Xu YJ, Fang WY (2013). Prevalence and spectrum of GATA5 mutations associated with congenital heart disease. Int J Cardiol.

[9] Granados-Riveron JT, Pope M, Bu’lock FA, Thornborough C, Eason J, Setchfield K (2012). Combined mutation screening of NKX2-5, GATA4, and TBX5 in congenital heart disease: multiple heterozygosity and novel mutations. Congenit Heart Dis.

[10] Wang J, Sun YM, Yang YQ (2012). Mutation spectrum of the GATA4 gene in patients with idiopathic atrial fibrillation. Mol Biol Rep.

[11] Li J, Liu WD, Yang ZL, Yuan F, Xu L, Li RG (2014). Prevalence and spectrum of GATA4 mutations associated with sporadic dilated cardiomyopathy. Gene.

[12] Jumppanen M, Kinnunen SM, Välimäki MJ, Talman V, Auno S, Bruun T (2019). Synthesis, identification, and structure-activity relationship analysis of GATA4 and NKX2-5 protein-protein interaction modulators. J Med Chem.

[13] Wang T, Chen L, Yang T, Huang P, Wang L, Zhao L (2019). Congenital heart disease and risk of cardiovascular disease: a meta-analysis of cohort studies. J Am Heart Assoc.

[14] Chen J, Qi B, Zhao J, Liu W, Duan R, Zhang M (2016). A novel mutation of GATA4 (K300T) associated with familial atrial septal defect. Gene.

[15] Suluba E, Shuwei L, Xia Q, Mwanga A (2020). Congenital heart diseases: genetics, non-inherited risk factors, and signaling pathways. Egypt J Med Hum Genet.

[16] Su W, Zhu P, Wang R, Wu Q, Wang M, Zhang X (2017). Congenital heart diseases and their association with the variant distribution features on susceptibility genes. Clin Genet.

[17] Kalayinia S, Maleki M, Rokni-Zadeh H, Changi-Ashtiani M, Ahangar H, Biglari A (2019). GATA4 screening in Iranian patients of various ethnicities affected with congenital heart disease: co-occurrence of a novel de novo translocation ( 5;7) and a likely pathogenic heterozygous GATA4 mutation in a family with autosomal dominant congenital heart disease. J Clin Lab Anal.

[18] Clark KL, Yutzey KE, Benson DW (2006). Transcription factors and congenital heart defects. Annu Rev Physiol.

[19] El Bouchikhi I, Bouguenouch L, Moufid FZ, Belhassan K, Samri I, Chaouti A (2020). Absence of GATA4 mutations in Moroccan patients with atrial septal defect (ASD) provides further evidence of limited involvement of GATA4 in major congenital heart defects. Eurasian J Med.

[20] Misra C, Sachan N, McNally CR, Koenig SN, Nichols HA, Guggilam A (2012). Congenital heart disease-causing GATA4 mutation displays functional deficits in vivo. PLoS Genet.

[21] Maitra M, Schluterman MK, Nichols HA, Richardson JA, Lo CW, Srivastava D (2009). Interaction of GATA4 and GATA6 with Tbx5 is critical for normal cardiac development. Dev Biol.

[22] Stenson PD, Ball EV, Mort M, Phillips AD, Shiel JA, Thomas NS (2003). Human Gene Mutation Database (HGMD): 2003 update. Hum Mutat.

[23] Landrum MJ, Lee JM, Benson M, Brown GR, Chao C, Chitipiralla S (2018). ClinVar: improving access to variant interpretations and supporting evidence. Nucleic Acids Res.

[24] Stelzer G, Rosen N, Plaschkes I, Zimmerman S, Twik M, Fishilevich S (2016). The GeneCards suite: from gene data mining to disease genome sequence analyses. Curr Protoc Bioinformatics.

[25] Karczewski KJ, Weisburd B, Thomas B, Solomonson M, Ruderfer DM, Kavanagh D (2017). The ExAC browser: displaying reference data information from over 60 000 exomes. Nucleic Acids Res.

[26] Auton A, Brooks LD, Durbin RM, Garrison EP, Kang HM, Korbel JO (2015). A global reference for human genetic variation. Nature.

[27] Karczewski KJ, Francioli LC, Tiao G, Cummings BB, Alföldi J, Wang Q (2020). The mutational constraint spectrum quantified from variation in 141,456 humans. Nature.

[28] Auer PL, Reiner AP, Wang G, Kang HM, Abecasis GR, Altshuler D (2016). Guidelines for large-scale sequence-based complex trait association studies: lessons learned from the NHLBI exome sequencing project. Am J Hum Genet.

[29] Brody JA, Morrison AC, Bis JC, O’Connell JR, Brown MR, Huffman JE (2017). Analysis commons, a team approach to discovery in a big-data environment for genetic epidemiology. Nat Genet.

[30] Fattahi Z, Beheshtian M, Mohseni M, Poustchi H, Sellars E, Nezhadi SH (2019). Iranome: a catalog of genomic variations in the Iranian population. Hum Mutat.

[31] Scott EM, Halees A, Itan Y, Spencer EG, He Y, Azab MA (2016). Characterization of Greater Middle Eastern genetic variation for enhanced disease gene discovery. Nat Genet.

[32] Sim NL, Kumar P, Hu J, Henikoff S, Schneider G, Ng PC (2012). SIFT web server: predicting effects of amino acid substitutions on proteins. Nucleic Acids Res.

[33] Adzhubei IA, Schmidt S, Peshkin L, Ramensky VE, Gerasimova A, Bork P (2010). A method and server for predicting damaging missense mutations. Nat Methods.

[34] Choi Y, Chan AP (2015). PROVEAN web server: a tool to predict the functional effect of amino acid substitutions and indels. Bioinformatics.

[35] Rentzsch P, Witten D, Cooper GM, Shendure J, Kircher M (2019). CADD: predicting the deleteriousness of variants throughout the human genome. Nucleic Acids Res.

[36] Schwarz JM, Cooper DN, Schuelke M, Seelow D (2014). MutationTaster2: mutation prediction for the deep-sequencing age. Nat Methods.

[37] Cooper GM, Stone EA, Asimenos G, Green ED, Batzoglou S, Sidow A (2005). Distribution and intensity of constraint in mammalian genomic sequence. Genome Res.

[38] Adzhubei I, Jordan DM, Sunyaev SR. Predicting functional effect of human missense mutations using PolyPhen-2. Curr Protoc Hum Genet 2013;Chapter 7:Unit7.20. 10.1002/0471142905.hg0720s76 PMC4480630 23315928

[39] Niroula A, Vihinen M (2019). How good are pathogenicity predictors in detecting benign variants?. PLoS Comput Biol.

[40] Huber CD, Kim BY, Lohmueller KE (2020). Population genetic models of GERP scores suggest pervasive turnover of constrained sites across mammalian evolution. PLoS Genet.

[41] Szklarczyk D, Franceschini A, Kuhn M, Simonovic M, Roth A, Minguez P (2011). The STRING database in 2011: functional interaction networks of proteins, globally integrated and scored. Nucleic Acids Res.

[42] Szklarczyk D, Gable AL, Lyon D, Junge A, Wyder S, Huerta-Cepas J (2019). STRING v11: protein-protein association networks with increased coverage, supporting functional discovery in genome-wide experimental datasets. Nucleic Acids Res.

[43] Venselaar H, Te Beek TA, Kuipers RK, Hekkelman ML, Vriend G (2010). Protein structure analysis of mutations causing inheritable diseases An e-Science approach with life scientist friendly interfaces. BMC Bioinformatics.

[44] Roy A, Kucukural A, Zhang Y (2010). I-TASSER: a unified platform for automated protein structure and function prediction. Nat Protoc.

[45] Yang J, Yan R, Roy A, Xu D, Poisson J, Zhang Y (2015). The I-TASSER Suite: protein structure and function prediction. Nat Methods.

[46] Yang J, Zhang Y (2015). I-TASSER server: new development for protein structure and function predictions. Nucleic Acids Res.

[47] Zhang Y (2008). I-TASSER server for protein 3D structure prediction. BMC Bioinformatics.

[48] Zhang W, Li X, Shen A, Jiao W, Guan X, Li Z (2008). GATA4 mutations in 486 Chinese patients with congenital heart disease. Eur J Med Genet.

[49] Jiang JQ, Shen FF, Fang WY, Liu X, Yang YQ (2011). Novel GATA4 mutations in lone atrial fibrillation. Int J Mol Med.

[50] Liu XY, Yang YQ, Ma J, Lin XP, Zheng JH, Bai K (2010). [Novel GATA4 mutations identified in patients with congenital atrial septal defects]. Zhonghua Xin Xue Guan Bing Za Zhi.

[51] Liu XY, Wang J, Zheng JH, Bai K, Liu ZM, Wang XZ (2011). Involvement of a novel GATA4 mutation in atrial septal defects. Int J Mol Med.

[52] Chen MW, Pang YS, Guo Y, Liu BL, Shen J, Song HD (2009). [Association between GATA-4 mutations and congenital cardiac septal defects in Han Chinese patients]. Zhonghua Xin Xue Guan Bing Za Zhi.

[53] Yang YQ, Wang J, Liu XY, Chen XZ, Zhang W, Wang XZ (2013). Mutation spectrum of GATA4 associated with congenital atrial septal defects. Arch Med Sci.

[54] Yang YQ, Li L, Wang J, Liu XY, Chen XZ, Zhang W (2012). A novel GATA4 loss-of-function mutation associated with congenital ventricular septal defect. Pediatr Cardiol.

[55] Yang YQ, Gharibeh L, Li RG, Xin YF, Wang J, Liu ZM (2013). GATA4 loss-of-function mutations underlie familial tetralogy of Fallot. Hum Mutat.

[56] Hirayama-Yamada K, Kamisago M, Akimoto K, Aotsuka H, Nakamura Y, Tomita H (2005). Phenotypes with GATA4 or NKX25 mutations in familial atrial septal defect. Am J Med Genet A.

[57] Yang YQ, Wang J, Liu XY, Chen XZ, Zhang W, Wang XZ (2012). Novel GATA4 mutations in patients with congenital ventricular septal defects. Med Sci Monit.

[58] Yang YQ, Tang YQ, Liu XY, Lin XP, Chen YH (2010). [A novel GATA4 mutation leading to congenital ventricular septal defect]. Zhonghua Yi Xue Yi Chuan Xue Za Zhi.

[59] Liu Y, Li B, Xu Y, Sun K (2017). Mutation screening of GATA4 gene in CTD patients within Chinese Han population. Pediatr Cardiol.

[60] Chen MW, Pang YS, Guo Y, Pan JH, Liu BL, Shen J (2010). GATA4 mutations in Chinese patients with congenital cardiac septal defects. Pediatr Cardiol.

[61] Wang E, Sun S, Qiao B, Duan W, Huang G, An Y (2013). Identification of functional mutations in GATA4 in patients with congenital heart disease. PLoS One.

[62] Butler TL, Esposito G, Blue GM, Cole AD, Costa MW, Waddell LB (2010). GATA4 mutations in 357 unrelated patients with congenital heart malformation. Genet Test Mol Biomarkers.

[63] Yang YQ, Wang MY, Zhang XL, Tan HW, Shi HF, Jiang WF (2011). GATA4 loss-of-function mutations in familial atrial fibrillation. Clin Chim Acta.

[64] Wang J, Hu DY, Li XM, Xin YF, Zhou H, Wang LJ (2010). [Novel GATA4 mutations identified in patients with congenital heart disease]. Zhonghua Yi Xue Za Zhi.

[65] Tomita-Mitchell A, Maslen CL, Morris CD, Garg V, Goldmuntz E (2007). GATA4 sequence variants in patients with congenital heart disease. J Med Genet.

[66] Porto MP, Vergani N, Carvalho AC, Cernach MC, Brunoni D, Perez AB (2010). Novel mutations in the TBX5 gene in patients with Holt-Oram syndrome. Genet Mol Biol.

[67] Yoshida A, Morisaki H, Nakaji M, Kitano M, Kim KS, Sagawa K (2016). Genetic mutation analysis in Japanese patients with non-syndromic congenital heart disease. J Hum Genet.

[68] Rajagopal SK, Ma Q, Obler D, Shen J, Manichaikul A, Tomita-Mitchell A (2007). Spectrum of heart disease associated with murine and human GATA4 mutation. J Mol Cell Cardiol.

[69] Peng T, Wang L, Zhou SF, Li X (2010). Mutations of the GATA4 and NKX25 genes in Chinese pediatric patients with non-familial congenital heart disease. Genetica.

[70] Shaker O, Omran S, Sharaf E, G AH, Mashaly M, N EAG (2017). A novel mutation in exon 1 of GATA4 in Egyptian patients with congenital heart disease. Turk J Med Sci.

[71] Mattapally S, Nizamuddin S, Murthy KS, Thangaraj K, Banerjee SK (2015). c620C > T mutation in GATA4 is associated with congenital heart disease in South India. BMC Med Genet.

[72] Reamon-Buettner SM, Borlak J (2005). GATA4 zinc finger mutations as a molecular rationale for septation defects of the human heart. J Med Genet.

[73] Nemer G, Fadlalah F, Usta J, Nemer M, Dbaibo G, Obeid M (2006). A novel mutation in the GATA4 gene in patients with tetralogy of Fallot. Hum Mutat.

[74] Lourenço D, Brauner R, Rybczynska M, Nihoul-Fékété C, McElreavey K, Bashamboo A (2011). Loss-of-function mutation in GATA4 causes anomalies of human testicular development. Proc Natl Acad Sci U S A.

[75] Posch MG, Boldt LH, Polotzki M, Richter S, Rolf S, Perrot A (2010). Mutations in the cardiac transcription factor GATA4 in patients with lone atrial fibrillation. Eur J Med Genet.

[76] Xiong F, Li Q, Zhang C, Chen Y, Li P, Wei X (2013). Analyses of GATA4, NKX25, and TFAP2B genes in subjects from southern China with sporadic congenital heart disease. Cardiovasc Pathol.

[77] Wang J, Li XM, Xin YF, Wang LJ, Xu WJ, Hu DY (2010). [Genetic screening for novel GATA4 mutations associated with congenital atrial septal defect]. Zhonghua Xin Xue Guan Bing Za Zhi.

[78] Li RG, Li L, Qiu XB, Yuan F, Xu L, Li X (2013). GATA4 loss-of-function mutation underlies familial dilated cardiomyopathy. Biochem Biophys Res Commun.

[79] Soheili F, Jalili Z, Rahbar M, Khatooni Z, Mashayekhi A, Jafari H (2018). Novel mutation of GATA4 gene in Kurdish population of Iran with nonsyndromic congenital heart septals defects. Congenit Heart Dis.

[80] Posch MG, Perrot A, Schmitt K, Mittelhaus S, Esenwein EM, Stiller B (2008). Mutations in GATA4, NKX25, CRELD1, and BMP4 are infrequently found in patients with congenital cardiac septal defects. Am J Med Genet A.

[81] Chen Y, Mao J, Sun Y, Zhang Q, Cheng HB, Yan WH (2010). A novel mutation of GATA4 in a familial atrial septal defect. Clin Chim Acta.

[82] El Malti R, Liu H, Doray B, Thauvin C, Maltret A, Dauphin C (2016). A systematic variant screening in familial cases of congenital heart defects demonstrates the usefulness of molecular genetics in this field. Eur J Hum Genet.

[83] Wang J, Fang M, Liu XY, Xin YF, Liu ZM, Chen XZ (2011). A novel GATA4 mutation responsible for congenital ventricular septal defects. Int J Mol Med.

[84] Garg V, Kathiriya IS, Barnes R, Schluterman MK, King IN, Butler CA (2003). GATA4 mutations cause human congenital heart defects and reveal an interaction with TBX5. Nature.

[85] Sarkozy A, Conti E, Neri C, D’Agostino R, Digilio MC, Esposito G (2005). Spectrum of atrial septal defects associated with mutations of NKX25 and GATA4 transcription factors. J Med Genet.

[86] Chen Y, Han ZQ, Yan WD, Tang CZ, Xie JY, Chen H (2010). A novel mutation in GATA4 gene associated with dominant inherited familial atrial septal defect. J Thorac Cardiovasc Surg.

[87] Zhang X, Wang J, Wang B, Chen S, Fu Q, Sun K (2016). A novel missense mutation of GATA4 in a Chinese family with congenital heart disease. PLoS One.

[88] D’Amato E, Giacopelli F, Giannattasio A, D’Annunzio G, Bocciardi R, Musso M (2010). Genetic investigation in an Italian child with an unusual association of atrial septal defect, attributable to a new familial GATA4 gene mutation, and neonatal diabetes due to pancreatic agenesis. Diabet Med.

[89] Kodo K, Nishizawa T, Furutani M, Arai S, Ishihara K, Oda M (2012). Genetic analysis of essential cardiac transcription factors in 256 patients with non-syndromic congenital heart defects. Circ J.

[90] Reamon-Buettner SM, Cho SH, Borlak J (2007). Mutations in the 3’-untranslated region of GATA4 as molecular hotspots for congenital heart disease (CHD). BMC Med Genet.

[91] Al-Azzouny MA, El Ruby MO, Issa HA, Behiry EG, Elsayed NR, Fayez AG (2016). Detection and putative effect of GATA4 gene variants in patients with congenital cardiac septal defects. Cell Mol Biol (Noisy-le-grand).

[92] Dinesh SM, Lingaiah K, Savitha MR, Krishnamurthy B, Narayanappa D, Ramachandra NB (2011). GATA4 specific nonsynonymous single-nucleotide polymorphisms in congenital heart disease patients of Mysore, India. Genet Test Mol Biomarkers.

[93] Wang J, Lu Y, Chen H, Yin M, Yu T, Fu Q (2011). Investigation of somatic NKX2-5, GATA4 and HAND1 mutations in patients with tetralogy of Fallot. Pathology.

[94] Bonachea EM, Zender G, White P, Corsmeier D, Newsom D, Fitzgerald-Butt S (2014). Use of a targeted, combinatorial next-generation sequencing approach for the study of bicuspid aortic valve. BMC Med Genomics.

[95] Suzuki YJ (2011). Cell signaling pathways for the regulation of GATA4 transcription factor: implications for cell growth and apoptosis. Cell Signal.

[96] Naghavi-Behzad M, Alizadeh M, Azami S, Foroughifar S, Ghasempour-Dabbaghi K, Karzad N (2013). Risk factors of congenital heart diseases: a case-control study in Northwest Iran. J Cardiovasc Thorac Res.

[97] Zhou P, He A, Pu WT (2012). Regulation of GATA4 transcriptional activity in cardiovascular development and disease. Curr Top Dev Biol.

[98] Fang T, Zhu Y, Xu A, Zhang Y, Wu Q, Huang G (2019). Functional analysis of the congenital heart disease-associated GATA4 H436Y mutation in vitro. Mol Med Rep.

[99] Sepulveda JL, Vlahopoulos S, Iyer D, Belaguli N, Schwartz RJ (2002). Combinatorial expression of GATA4, Nkx2-5, and serum response factor directs early cardiac gene activity. J Biol Chem.

[100] Garnatz AS, Gao Z, Broman M, Martens S, Earley JU, Svensson EC (2014). FOG-2 mediated recruitment of the NuRD complex regulates cardiomyocyte proliferation during heart development. Dev Biol.

[101] Pizzuti A, Sarkozy A, Newton AL, Conti E, Flex E, Digilio MC (2003). Mutations of ZFPM2/FOG2 gene in sporadic cases of tetralogy of Fallot. Hum Mutat.

[102] De Luca A, Sarkozy A, Ferese R, Consoli F, Lepri F, Dentici ML (2011). New mutations in ZFPM2/FOG2 gene in tetralogy of Fallot and double outlet right ventricle. Clin Genet.

[103] Tan ZP, Huang C, Xu ZB, Yang JF, Yang YF (2012). Novel ZFPM2/FOG2 variants in patients with double outlet right ventricle. Clin Genet.

[104] Qiao XH, Wang F, Zhang XL, Huang RT, Xue S, Wang J (2017). MEF2C loss-of-function mutation contributes to congenital heart defects. Int J Med Sci.

[105] Ieda M, Fu JD, Delgado-Olguin P, Vedantham V, Hayashi Y, Bruneau BG (2010). Direct reprogramming of fibroblasts into functional cardiomyocytes by defined factors. Cell.

[106] Li FF, Deng X, Zhou J, Yan P, Zhao EY, Liu SL (2016). Characterization of human bone morphogenetic protein gene variants for possible roles in congenital heart disease. Mol Med Rep.

[107] Song W, Wang H, Wu Q (2015). Atrial natriuretic peptide in cardiovascular biology and disease (NPPA). Gene.

[108] Reamon-Buettner SM, Borlak J (2006). HEY2 mutations in malformed hearts. Hum Mutat.

[109] Garcia-Blanco MA, Baraniak AP, Lasda EL (2004). Alternative splicing in disease and therapy. Nat Biotechnol.

[110] Reamon-Buettner SM, Borlak J (2004). TBX5 mutations in non-Holt-Oram syndrome (HOS) malformed hearts. Hum Mutat.

[111] Reamon-Buettner SM, Hecker H, Spanel-Borowski K, Craatz S, Kuenzel E, Borlak J (2004). Novel NKX2-5 mutations in diseased heart tissues of patients with cardiac malformations. Am J Pathol.

[112] Reamon-Buettner SM, Borlak J (2004). Somatic NKX2-5 mutations as a novel mechanism of disease in complex congenital heart disease. J Med Genet.

